# Adenoviral fiber-knob based vaccination elicits efficient neutralizing antibodies and T cell responses against adenovirus infection

**DOI:** 10.1186/s12985-024-02520-w

**Published:** 2024-10-07

**Authors:** Ahmed Orabi, Kamyar Shameli, Ulrike Protzer, Hassan Moeini

**Affiliations:** 1 Institute of Virology, School of Medicine, Technical University of Munich, Munich, Germany; 2 Department of Virology, Faculty of Veterinary Medicine, Zagazig University, Zagazig, Egypt; 3 Institute of Virology, Helmholtz Munich, Munich, Germany; 4 German Center for Infection Research (DZIF), Munich partner Site, Munich, Germany

**Keywords:** Adenoviral vaccination, Fiber-knob, Neutralizing antibodies, CD4^+^ T cell responses, Adenovirus infection

## Abstract

**Background:**

Human adenoviruses (HAdVs) frequently cause common respiratory or gastrointestinal infections among children, adults, individuals with immune deficiencies, and other vulnerable populations with varying degree of symptoms, ranging from mild to server, and in some cases, even fatalities. Despite the significant clinical impact of HAdVs, there is currently no approved vaccine available.

**Methods:**

This study explores the potential of the adenovirus type 5 fiber knob (Ad5-FK) to stimulate the production of Ad-specific neutralizing antibodies and T-cell responses in mice. Based on structure predictions, we first expressed Ad5-FK in *E. coli* and confirmed the assembly of FK into its trimeric form. After testing the binding capability of the trimeric FK to susceptible cells, the immunogenicity of the protein in combination with the c-di-AMP adjuvant was assessed in BALB/c mice.

**Results:**

The purified Ad5-FK exhibited self-trimerization and maintained correct conformation akin to the authentic FK structure. This facilitated effective binding to susceptible HEK293 cells. Notably, the protein demonstrated significant inhibition of HEK293 cells infection by rAd5-GFP. Immunization of BALB/c mice with Ad5-FK, or Ad5-FK mixed with c-di-AMP yielded FK-specific antibodies with potent neutralization capacity. Significantly, Ad5-FK was found to elicit a vigorous CD4^+^ T-cell response in the immunized mice.

**Conclusion:**

Our findings underscore the efficacy of FK-based vaccine in eliciting anti-Ad humoral immune response and CD4 T-cell immune reactions essential for protection against viral infections.

**Supplementary Information:**

The online version contains supplementary material available at 10.1186/s12985-024-02520-w.

## Background

Human adenoviruses (HAd) cause common clinical diseases including acute respiratory, digestive, cardiac and ocular clinical diseases [1, 2], which are mostly self-limiting or mild. However, in some cases, infected patients may experience severe outcomes and require intensive care. Tragically, in more dire situations, infection can lead to disability or even loss of life [3]. The high-risk groups for adenoviral infections are particularly immunocompromised individuals and children, especially those younger than 5 years old [4]. While most adenoviral infections are self-limited, they can be severe and even fatal for immunocompromised patients. In rare cases, even previously healthy patients can succumb to the infection [2, 5]. During the years 2021–2022, concerning trend was observed as numerous previously healthy children were diagnosed with acute hepatitis and HAd viremia [6]. Additionally, potentially fatal outbreaks caused by HAd 14 were recorded in residential facilities and military bases [7]. These outbreaks can impose a significant burden, both financially and operationally, with significant medical expenses. Vaccination has proven to be a cost effective and indispensable strategy in preventing viral infections. Although the efficacy of the live adenovirus vaccine prepared for the US military in 1971 has been demonstrated, the development of new adenovirus vaccines remains necessary due to concerns over positive viral shedding and unexpected higher incidence of adverse effects [8, 9], as well as the potential risks posed by new emerging virus infection [6]. Adenovirus fiber-knob (Ad-FK) plays a crucial role in attaching the virus to susceptible cells, including those with the Coxsackie B virus and Adenovirus receptor (CAR) [10]. Comprising multiple immunogenic epitopes, Ad-FK is the primary target for neutralizing antibodies (NAbs) resulting from natural infection [11]. In the present study, we produced a highly pure adenovirus 5 fiber-knob (Ad5-FK) in *E. coli* and investigated its capacity to induce the production of neutralizing antibodies and T-cell responses in mice.

## Materials and methods

### Adenovirus Fiber Knob expression and purification

**Fig. 1:**
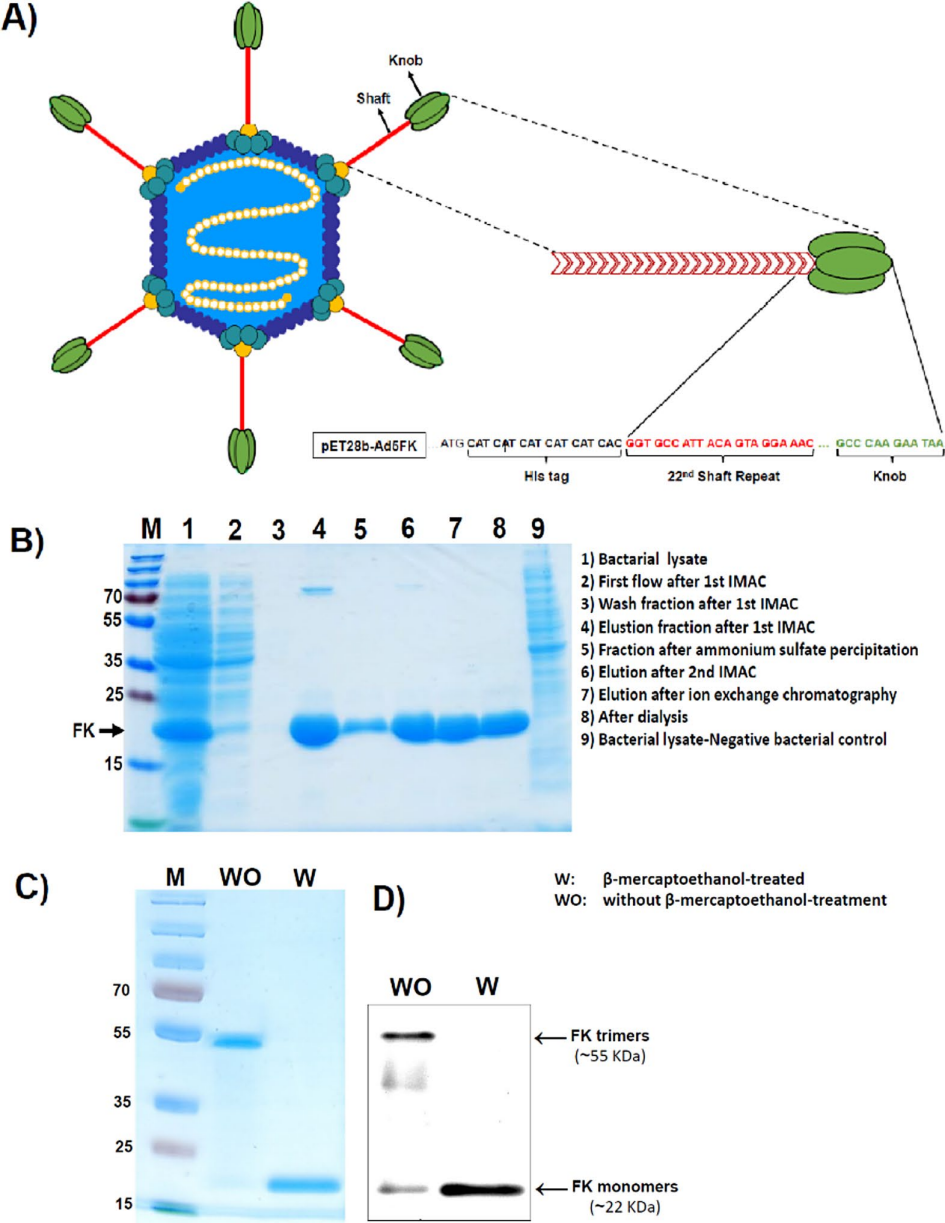
**(A)** Schematic diagram of adenovirus Fiber-Knob (FK)-based vaccine construct. The construct includes the knob part of the protein, the final (22nd ) shaft repeat of the adenoviral fiber and a 6-His tag at the N-terminal required for protein purification. **(B)***E. coli*-expressed Ad5-FK (~ 22 KDa) on denaturing SDS-PAGE: FK protein was efficiently produced as a soluble protein with molecular weight of approximately 22 KDa. The purification process of the protein involved several steps including two consecutive IMAC steps using Ni-NTA columns (lanes 2–4 and 6), an intermediate step of ammonium sulfate precipitation (lane 5) and a final purification step using ion exchange chromatography (lane 7). **(C)** Native and denatured Ad5-FK on Coomassie Blue-stained SDS-PAGE gel. Purified FK without β-mercaptoethanol treatment (WO) was found to self-assemble into a trimer form of ~ 55 KDa, while no trimerization was detected in the β-mercaptoethanol-treated sample (W). **(D)** Western blot of native and denatured Ad5-FK using anti-His tag antibody. The trimerization pattern in the FK sample not treated with β-mercaptoethanol (WO), as observed on SDS-PAGE, was also confirmed by Western blotting

The Ad5-FK gene fragment was amplified from pAD-Ad5 vector harboring adenovirus 5 genome as a template using the Phusion™ High-Fidelity DNA Polymerase and a pair of primers 5ʹTAAGAAGGAGATATAATGCATCATCATCATCATCACGGTGCCATTACAGTAGGAAACʹ3 and 5ʹGTGGTGGTGGTGGTGCTCGAGTTATTCTTGGGCAATGTATGAAAAʹ3. The forward primer included a 6-His tag and the oligonucleotide sequences encoding the first seven residues of the Ad5-22nd shaft repeat (GAITVGN), while the reverse primer included oligonucleotide sequences encoding the last six residues of the knob domain, SYIAQE (Fig. 1A) [12]. The amplified fragment was gel purified and inserted into the expression vector pET-28b downstream of T7 promoter using the In-Fusion Snap Assembly kit (Takara Bio, USA). The clones were screened by restriction enzyme digestion; the correct orientation and sequence of the insert gene were then verified with the double-strand sequencing. The recombinant pET28b-FK plasmid was introduced into the competent *E. coli* BL21 (DE3) cells for the expression of the Ad5-FK. The FK protein was purified by a two-step immobilized metal affinity chromatography (IMAC) approach using the Thermo Scientific HisPur Ni-NTA Purification Kit (Thermo Fisher Scientific, USA). Initially, the column was equilibrated using the equilibration buffer, and the protein sample was loaded onto the column. Thereafter, the captured proteins were eluted using the elution buffer (50 mM Tris-HCl, 0.1 M NaCl, 300 mM imidazole, pH 8.5) and then precipitated with 100% ammonium sulphate at 4 °C. After desalting using the Zeba Spin Desalting Column (7 K MWCO, Thermo Fisher Scientific, USA) with a buffer of 20 mM Tris-HCl at pH 8, the FK protein was refined using a 5-ml HiTrap TM QXL column (Cytiva). Initially, the column was equilibrated using ion-exchange buffer (20 mM Tris-HCl, pH 8). Next, the protein solution was injected onto the column for purification. The elution of FK was carried out with a linear gradient of elution buffer (20 m M Tris-HCl, 0.5 M NaCl, 1 mM EDTA, pH 7). Subsequently, the protein-containing fractions were pooled, concentrated through 100% ammonium sulfate precipitation, and then dialyzed against the final buffer (20 m M Tris-HCl, 1 mM EDTA, pH 8). The purified FK protein was validated on SDS-PAGE using Coomassie Brilliant Blue staining and by western blot using the HRP-conjugated anti-His tag monoclonal antibody (Thermo Scientific, USA). The concentration of the purified protein was measured using the Pierce BCA Protein Assay Kit (Thermo Fisher Scientific, USA) in accordance with the manufacturer’s instructions. Synthesized N-His tagged last (22nd ) shaft repeat (SR; GAITVGN) of Ad5-fiber was used as a negative control in the experiments.

### In vitro characterization of Ad5-FK by Electron Microscopy Visualization

For Electron Microscopy (EM) visualization and size determination, the purified Ad5-FK was subjected to Cryo-Electron Microscopy (Cryo-EM) analysis. The size of FK trimers (*n* = 120) was initially measured using the Image j software. Subsequently, the final size and distribution of the trimers was statistically determined using the SPSS-16 software.

### Interaction of Ad5-FK with Cell receptors

To assess the ability of the recombinant FK to bind to the receptor of adenovirus susceptible cells, the binding between the purified Ad5-FK and HEK293 cells was visualized by fluorescence microscopy. To this end, on the Ibidi USA µ-Slide 8 Well (Ibidi, USA), HEK293 cells were incubated with the purified Ad5-FK at a concentration of 0.5 µg/ml at RT for 30 min. After three washes with 1×PBS, the cells were treated with the FITC-conjugated anti-His-tag Monoclonal Antibody (AD1.1.10; Thermo Fisher Scientific, USA) at a concentration of 5 µg/ml for 1 h at 37 °C. Following the antibody treatment, the cells were washed (×3) with 1×PBS, and subsequently were mounted using a mounting solution including DAPI stain before being examined under the fluorescence microscope CKK41 (Olympus, Germany) at a magnification power of 63×. Adenovirus SR peptide was served as a negative control for the assay.

### Inhibition of Ad5 infection in HEK293 cells

Competitive inhibition of the recombinant Ad5-FK protein with the binding of HAd5V to cell receptors was tested in susceptible HEK293 cells. To this end, the cells were initially incubated with 11 different concentrations (5, 10, 20, 30, -100 ng/ml) of the purified FK at RT for 30 min. Following washing with 1×PBS, the cells were treated with recombinant replication-deficient adenovirus harboring the GFP reporter gene (rAd5-GFP), which was previously constructed in our lab, at an MOI of 5 for 45 min at RT. Although the rAd5-GFP virus is replication-deficient in normal cells due to deletion in the E1 region, the use of HEK293 cells, which stably express the E1A and E1B genes, allows for effective virus propagation in these cells. Following three washes with 1×PBS, the cells were incubated at 37 °C for an additional 18 h. The expression of the reporter GFP gene was then assessed by FACS using CytoFLEX Flow Cytometer (Beckman Coulter, USA). The adenovirus SR peptide was used as a negative control for the assay.

### Mouse immunization

Eight-week-old BALB/c mice with a weight of 20–25 g (4 per group) were intramuscularly injected with the FK (50 µg), FK (50 µg) plus 10 µg c-di-AMP (InvivoGen, USA) as adjuvant, or PBS as negative control three times with 2 weeks’ interval. Two weeks after the last injection, the mice were sacrificed, serum samples and spleens were collected for the determination of anti-FK antibody titer and T-cell response analysis, respectively.

### Antibody titration by FK-Based ELISA

FK-specific antibody concentrations were measured in mouse sera using a quantitative enzyme-linked immunosorbent assay (ELISA). To this end, ELISA plates were coated with 1 µg/ml FK overnight at 4⁰C. Two-fold serial dilutions of mouse IgG in 1×PBS, starting from 500 ng/ml, was used for the generation of a standard curve and quantitation of IgG concentration in the sera. After blocking the wells with 5% skimmed milk in 1×PBS for 2 h at RT, a 1 to 1000 dilution of sera in 1×PBS was added into the wells followed by 1.5 h incubation at RT. The wells were washed (×5) with washing buffer (1X PBS, 0.05% Tween 20) and then were treated with HRP-conjugated anti-mouse secondary antibodies for 1 h at RT. After five washing steps, the TMB substrate solution (Life technologies, USA) was applied to the wells; after 5 min incubation in dark at RT, the reaction was stopped with 0.16 M H_2_SO_4_, and absorbance values were measured at 450 nm using the Infinite F200 ELISA reader (Tecan, Germany). Antibody titers were finally measured using the *IgG* standard curve.

### Virus neutralization assay

Inhibition of Ad5 infection in HEK293 cells was used to determine neutralization activity of anti-adenovirus antibodies produced in the vaccinated mice. Sera sample dilutions from mice groups were tested for their ability to inhibit adenovirus infection. HEK 293 cells were seeded at 2 × 10^5^ cells per well in 96-well plates a day before the neutralization assay to reach 90–100% confluence. A fixed concentration (MOI = 1) of rAd5-GFP virus was incubated for 1 h at 37 °C either alone or with serial dilutions of sera (Six 2-fold serial dilutions for the serum samples starting at a 1:20). After incubation, 200 µl of each sample were applied to the cells and incubated for further 1 h at 37 °C to allow virus adsorption then the supernatant replaced with 200 µl of fresh 10% FBS DMEM. Finally, the GFP signals were recorded in each well after 24 h then the 100% neutralization titer was calculated. The 100% neutralization titer was recorded as the highest dilution of serum which yields no (0%) GFP signals in all infected wells (*n* = 5) relative to GFP signals in all wells (100%) treated with the virus alone at MOI = 1.

### T-cell reactivation assay

Splenocytes-associated lymphocytes were isolated from the spleens of vaccinated mice as previously described [13]. The isolated cells were then stimulated with Ad5-FK overlapping 15-mer peptides overnight, along with 1 µg/ml Brefeldin A (Sigma-Aldrich, Germany). Cells were live/dead-stained with ethidium monoazidebromide (Invitrogen, USA). For surface T-cell marker analysis anti-CD8α (Pacific Blue-conjugated) and anti-CD4 (PE-conjugated) (eBiosciences, Germany) antibodies were applied. Intracellular cytokine staining (ICS) was performed using FITC anti-IFNγ, PE-Cy7 anti-TNFα and APC anti-IL-2 antibodies (eBiosciences, Germany) with help of the Cytofix/Cytoperm kit (BD Biosciences, Germany). Flow Cytometry data were acquired using CytoFLEX S, (Beckman Coulter, USA) and analysed with FlowJo software (Treestar, USA).

### Statistical analysis

The data were analyzed by one-way ANOVA using the GraphPad Prism 9.5.0, and statistical significance was set at *p* < 0.05. The results were presented as means ± standard deviation (SD).

## Results

### Expression, purification and characterization of Ad5-FK

**Fig. 2:**
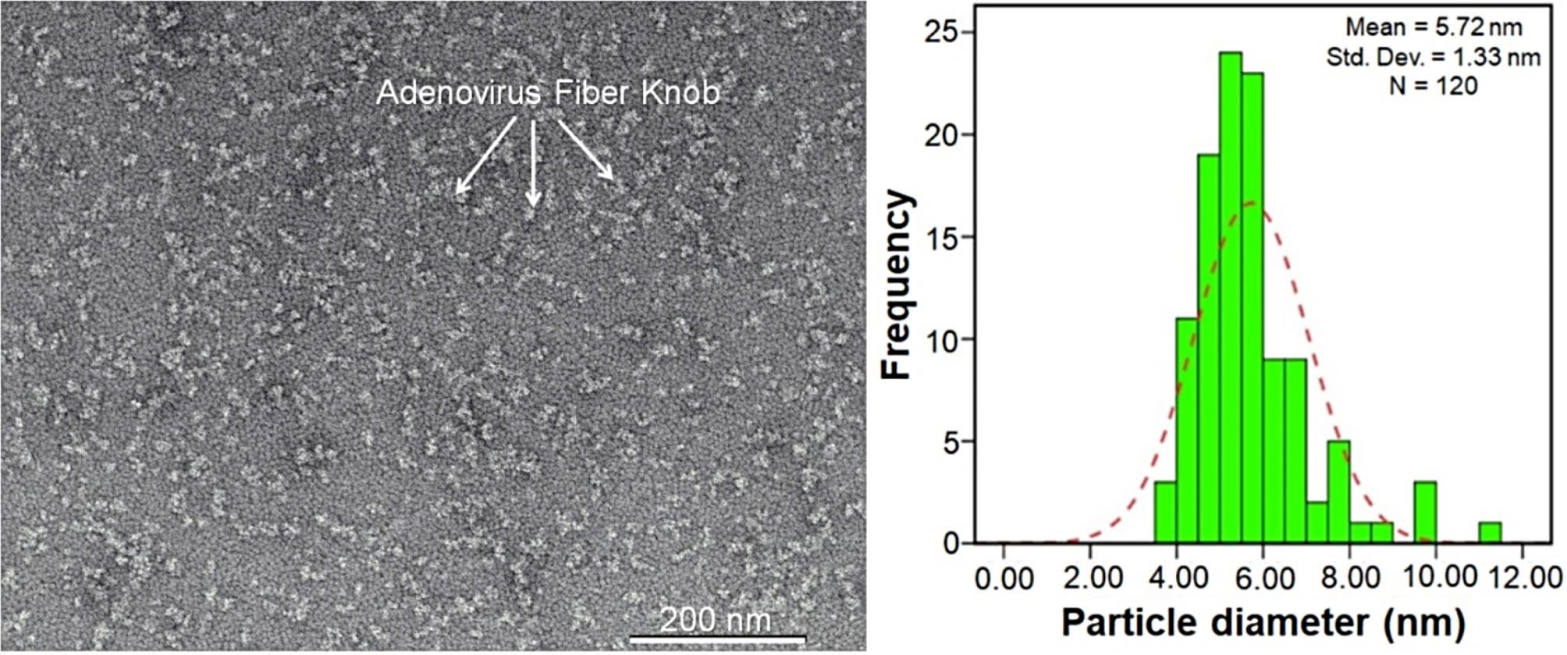
Purified Ad5-FK trimers images via Cryo-EM, exhibit sizes distribution diagram with approximately 5.72 ± 1.33 nm

The fiber knob domain of the Ad5-fiber protein, consisting of the final fiber shaft repeat (22nd ) and a 6-His tag at the N-terminal (Fig. 1A), was successfully amplified by PCR and subsequently cloned into the expression vector pET-28b, downstream of the T7 promoter. Sequencing of the vector at the insertion site confirmed the integrity of the target sequence. Upon expression in *E. coli* BL21 (DE3) cells, the FK protein was efficiently produced as a soluble protein with molecular weight of approximately 22 KDa, as confirmed by SDS-PAGE (Fig. 1B). The purification process of the protein involved several steps. Initially, the supernatant of bacterial lysate was subjected to two consecutive IMAC steps using Ni-NTA columns, with an intermediate step of ammonium sulfate precipitation. A final purification step was carried out using ion exchange chromatography. During the purification process, a small fraction of the protein was lost in the first flow-through fraction after the first IMAC step (Fig. 1B, lane 2); no detectable FK was found in the subsequent washing fractions (Fig. 1B, lane 3). Furthermore, after the 1st IMAC step, some larger protein impurities with molecular weight above 70 KDa co-eluted with FK (Fig. 1B, lane 4). However, these impurities were significantly reduced in the elution fraction after the 2nd IMAC step (Fig. 1B, lane 6) and were eliminated after the ion exchange chromatography (Fig. 1B, lane 7). Successful self-trimerization of FK monomers (~ 22 KDa) was evidence on SDS-PAGE gel (Fig. 1C) and Western blotting (Fig. 1D) with the weight of approximately ~ 55 KDa. The FK trimers, with a size of 5–6 nm, were also visualized by Cryo-EM (Fig. 2).

**Fig. 3:**
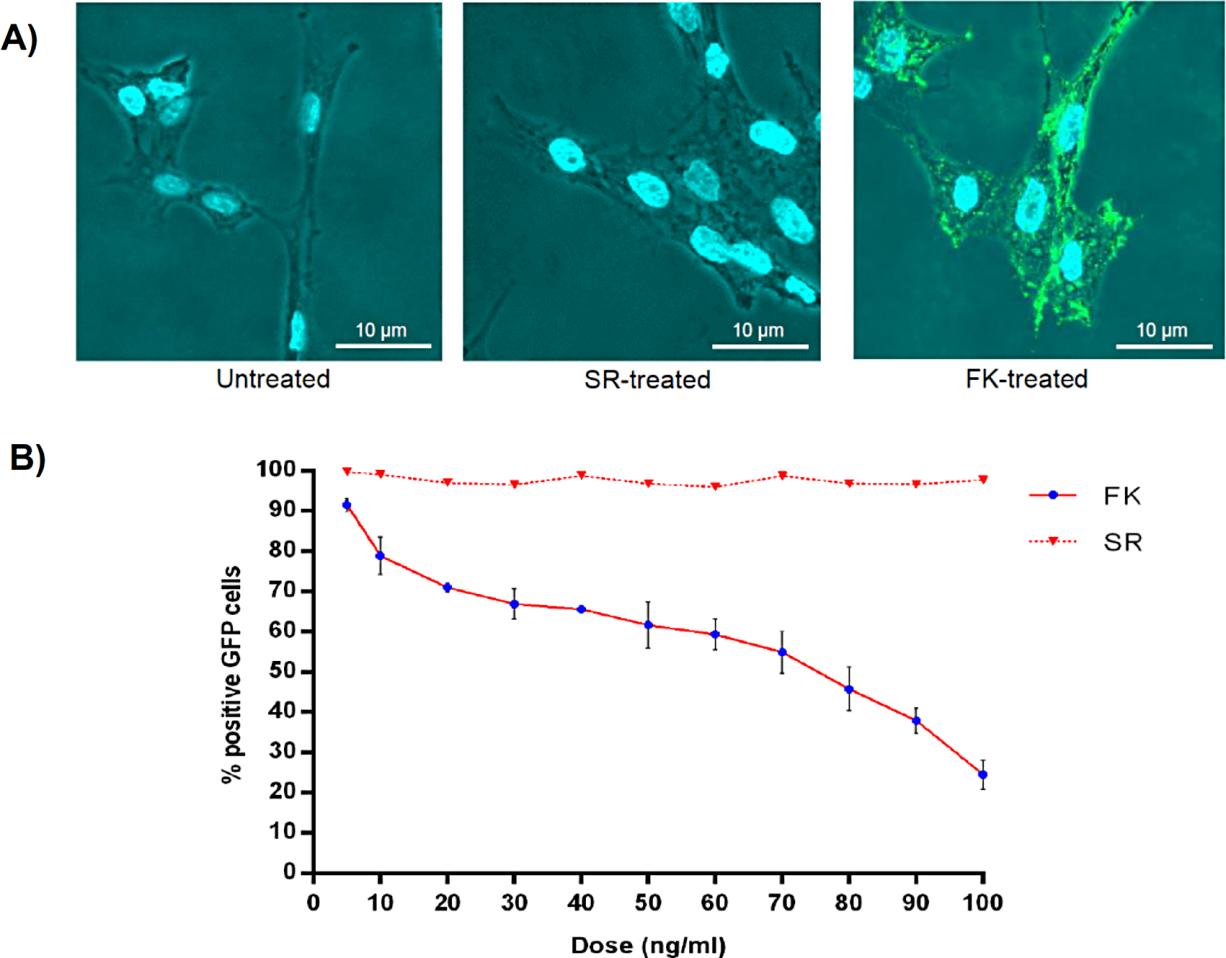
**(A)** Fluorescence microscopy visualization of Ad5-FK interaction with HEK293 cells. After treating HEK293 cells with purified FK protein, followed by incubation with FITC-conjugated anti-His tag antibody and DAPI stain, the interaction between FK and cell surface was visualized as distinct green dots surrounding HEK293 cells with blue-stained nuclei under fluorescence microscope at 40× magnification. **(B)** Competition of Ad5-FK with human adenovirus in binding to HEK293 cells. Following pre-incubation with different concentrations (5, 10, 20, 30, 100 ng/ml) of FK or SR proteins, cells were treated with rAd5-GFP at MOI 5. Eighteen hours later, virus infection was monitored by FACs through the expression of the reporter *gfp* gene. FK demonstrated the ability to reduce cell infection with the virus by up to 85% at a concentration of 100 ng/ml. In contrast, the negative control (SR) exhibited no inhibitory effect

The binding ability of the purified FK to adenovirus susceptible HEK293 cells was tested. The outcome indicated the successful binding of Ad-FK to the adenovirus susceptible cells, as evidenced by high fluorescence signals observed on the surface of the treated cells (Fig. 3A) in comparison to the negative SR control, where very low or no signals were detected. To investigate whether Ad-FK can compete with human adenovirus for binding to HEK293 cells, a series of experiments were conducted. HEK293 cells were pre-treated with different concentrations of FK and SR (as negative control). Subsequently, the cells were infected with rAd5-GFP. The percentage of infected cells was then assessed by FACS, where the SR pre-treated cells exhibited minimal variation in infection compared to the infected non-treated cells, indication of a lack of significant inhibition by SR. However, a progressive linear inhibition of the infection was detected in the FK pre-treated cells. As shown in Fig. 3B, at 5 ng/ml of Ad5-FK, virus infection was suppressed by approximately 10%. As the concentration of the FK increased, the inhibition percentage gradually rose, reaching around 85% inhibition at 100 ng/ml. This observation suggests that FK could effectively competes with the infectious virus for binding to the cell receptors, leading to a dose-dependent reduction in virus infection.

### Ad5-FK elicited efficient Adenovirus neutralizing antibodies and specific CD4 T-cell responses in mice

**Fig. 4:**
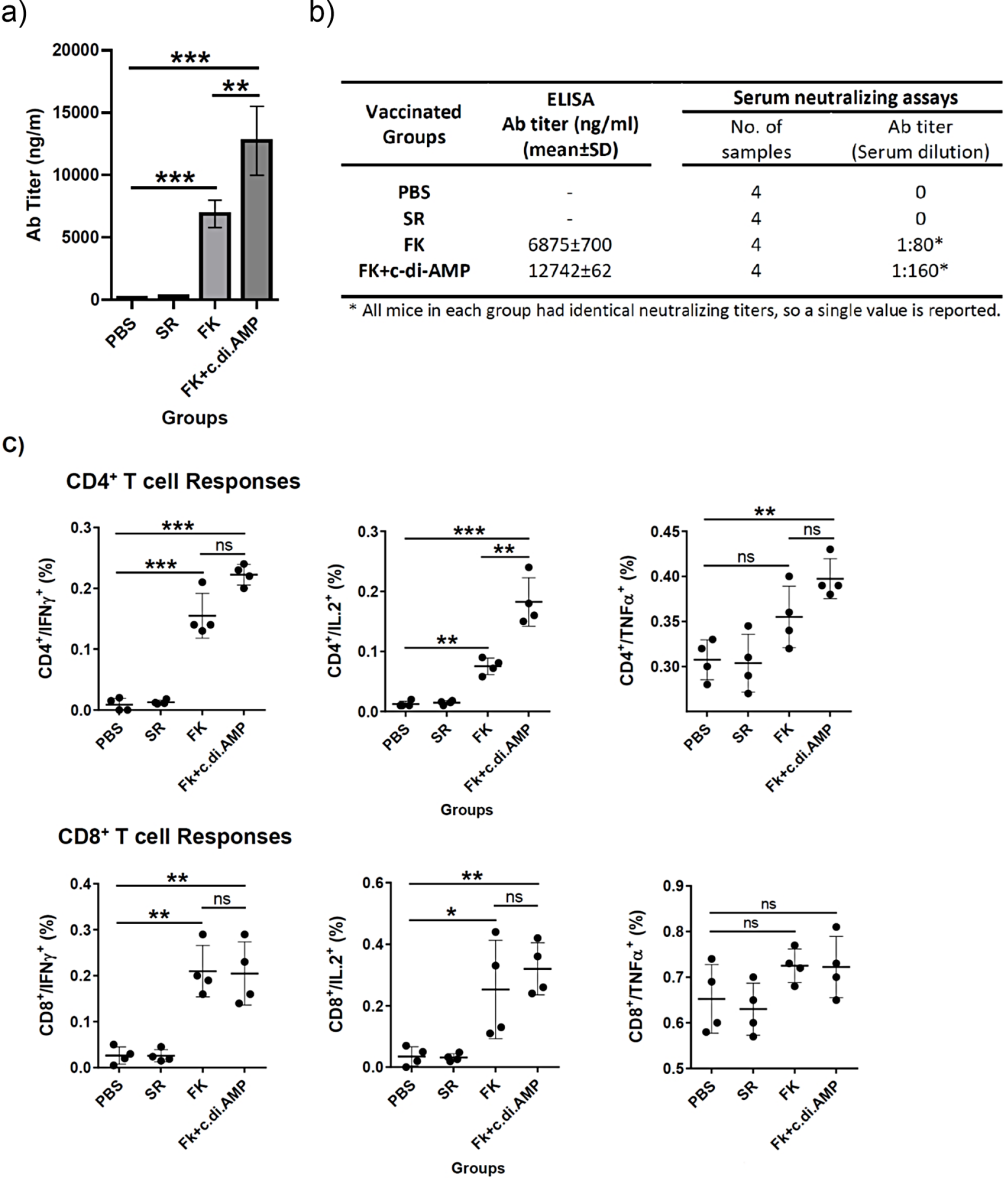
Evaluation of immune responses to Ad5-FK in BALB/c mice. Animals were injected with Ad5-FK alone or in combination with c-di-AMP adjuvant intramuscularly three times. Two weeks after the final injection, immune responses to the Ad5-FK were assessed. **(A)** Serum *IgG* titer by ELISA **(B)** Infection-neutralizing antibody titer against reAd5-GFP determined in mouse sera. **(C)** FK-specific CD4^+^ and CD8 T cell responses determined by ICS and FACS. *, *P* < 0.05; **, *P* < 0.01; ***, *P* < 0.001

To assess the immunogenicity of the Ad5-FK, BALB/c mice were intramuscular injected with Ad5-FK alone or Ad5-FK supplemented with c-di-AMP adjuvant three times with two weeks interval between injections. Two weeks after the final immunization, serum antibody and T-cell responses were evaluated. As depicted in Fig. 4A, both vaccinated groups exhibited a high level of FK-specific *IgG* antibodies compared to the control mice injected with PBS. The c-di-AMP adjuvant was found to significantly enhance the antibody response to the target antigen.

Evaluation of the neutralization activity of FK-specific antibodies, as illustrated in Fig. 4B, revealed that sera from mice vaccinated with Ad5-FK alone exhibited a neutralization titer (NAT) of 1:80, indicating that the elicited antibodies effectively inhibited virus infection at this dilution. In comparison, sera from mice vaccinated with Ad5-FK plus c-di-AMP adjuvant showed a higher neutralization titer of 1:160, demonstrating that the adjuvant significantly improved the neutralization efficacy of the antibodies. No neutralization was observed in the negative control group treated with the virus alone. Consistent with previous studies [12], these results confirm that FK-specific antibodies can effectively neutralize adenovirus infection by preventing virus attachment to target cells, and that the c-di-AMP adjuvant significantly enhances this neutralizing activity.

To evaluate the T cell responses triggered by our vaccine candidate, mice splenocytes were subjected to ex-vivo stimulation using a pool of overlapping FK peptides, followed by intracellular cytokine staining. This approach allowed us to determine the FK-specific cytokine-producing T cell population. Our results, summarized in Fig. 4C, revealed that, while the percentage of CD4^+^ and CD8^+^ T cells remained constant, FK elicited both specific CD4^+^ and CD8^+^ T cell responses in vaccinated mice. Notably, the inclusion of c-di-AMP as an adjuvant was found to enhance FK-induced CD4^+^ T cell responses and neutralizing antibody responses, as shown in Fig. 4B and C. However, c-di-AMP failed to improve FK-specific CD8^+^ T-cell responses (Fig. 4C). Deposit this, c-di-AMP demonstrated the ability to boost FK-specific CD4^+^ T-cell responses in vaccinated mice (Fig. 4C). Taken together, formulation of Ad5FK with c-di-AMP adjuvants induced strong FK-specific humoral and T cell immune responses.

## Discussion

Over the past few decades, adenoviruses have been widely used as vectors for therapeutic and immunization purposes, especially for diseases of apparent higher significance due to the self-limiting nature of many adenoviral infections. While vaccination has demonstrated effectiveness in preventing adenoviral infection, only one oral live adenovirus vaccine is currently available; however, its usage is restricted to the U.S. military. Nevertheless, there are apprehensions regarding positive viral shedding and unexpected higher occurrence of adverse effects [8, 9]. It is important to note that many outbreaks of adenoviral infections have not been limited to high-risk groups including immunocompromised individuals and children, they have also affected healthy individuals [14–18]. Some of these outbreaks resulted in significant mortality rates among healthy children, as seen in the recent outbreak in 2022 [6] and even adults [7, 19]. Given the favorable safety profile and efficacy demonstrated by subunit vaccines, even for immunocompromised patients [20, 21], our study aimed to explore the potential of adenoviral fiber-knob as an anti-adenoviral subunit vaccine. In addition to the hexon and penton proteins of adenoviral capsid, the fiber, particularly the fiber-knob, contains immunogenic epitopes capable of eliciting potent neutralizing antibodies in both mice and humans [22, 23]. Herein, we successfully expressed Ad5-FK in *E. coli* BL21 cells. The purification process involved two steps of IMAC and a final step of IEC with intermediate steps of ammonium sulfate precipitation, desalting and dialysis. This optimized purification strategy resulted in a highly purified Ad-FK (> 99%). Notably, this yield surpassed that obtained in previous studies using other *E. coli* strains [12] or the insect Sf9 cells [24]. The purified Ad5-FK was observed to self-trimerize and exhibited correct conformation with a consistent size of 5–6 nm, mirroring the authentic FK [25, 26]. The trimeric arrangement of monomers in the knob expressed in *E. coli* has been also reported by others [12, 27]. The existence of the knob in solution as a trimer suggests that the individual monomers self-associate in the absence of 21 of the 22 repeating protein motifs of the shaft normally found in the Ad5 fiber. The data are consistent with the possibility that the trimeric assembly of the fiber protein is energetically driven by the association of the individual globular subunits of the knob, as previously predicted [28]. Proper conformation of FK allowed efficient binding to susceptible HEK293 cells. Moreover, the purified Ad5-FK demonstrated significant inhibition of HEK293 cells infection with rAd5-GFP. Importantly, this inhibition was specific, as indicated by the nearly 0% inhibition observed in the negative control, SR. Furthermore, the inhibitory effect was concentration-dependent, consistent with the findings reported by Henry et al. [12]. Immunization with Ad5-FK, either alone or in combination with c-di-AMP adjuvant, elicited a significant level of FK-specific antibodies in the mice. While we acknowledge that a larger sample size and repeat experiments would strengthen the conclusions, the observed robust antibody production in this preliminary study indicates the high immunogenicity of the Ad5-FK as a vaccine candidate. As expected, the antisera from the mice immunized with adjuvanted FK exhibited stronger antibodies titer and neutralizing activity against the virus than the antisera from the FK-immunized mice. It has been shown that c-di-AMP promotes humoral as well as cellular immune responses to vaccine antigens in immunized mice [29]. In vitro re-stimulation of splenocytes from mice immunized with antigen in the presence of c-di-AMP has been shown to have stimulatory effect on dendritic cells (DCs), leading to T cell activation [30]. The prevalence of a CD4^+^ T-cell–driven response aligns with previous findings indicating that the majority of IFN-γ-secreting cells responsive to adenovirus in healthy individuals are CD4^+^ T cells [31]. Furthermore, in vitro models illustrate that CD4^+^ T-cell clones capable of recognizing antigen from the hexon protein have the capacity to induce lysis in infected target cells [32]. In line with this, among pediatric hematopoietic stem cell transplantation (HSCT) recipients, a delayed CD4 reconstitution is linked to increased susceptibility to adenoviral infection. Notably, a substantial proportion of patients who did not manifest adenoviremia following HSCT exhibited CD4-mediated anti-adenoviral responses within three months after transplantation [33, 34]. Importantly, our vaccine also exhibited the capability to generate robust CD4^+^ T-cell responses, which are necessary for optimal responses by other lymphocytes and stimulating B cell antibody production [35]. Regrettably, no suitable permissive animal model for the replication and infection of wild-type adenovirus has been established. Consequently, infected mice exhibit no discernible symptoms, such as elevated body temperature or weight loss. Furthermore, the viral load in the lungs of infected mice remains low and decreases over the course of prolonged infection. As a result, the need is pressing to develop a permissive animal model for human Ad infection and for evaluating vaccine candidates in future research.

## Conclusions

The results of this investigation demonstrate that the FK-based vaccine effectively induces a robust humoral immune response and elicits crucial T-cell immune reactions, which are important for protection against viral infections. While our study focuses on the fiber knob (FK) of Ad5, further research, including the analysis of fiber knob sequence identity across known adenovirus types and evaluating T-cell epitope conservation, is needed to directly test cross-reactivities and validate the efficacy of this approach against a broader range of adenovirus serotypes. This study lays the groundwork for future investigations into combinational vaccines that aim to provoke strong immune responses against various HAdV serotypes, potentially enhancing defense mechanisms against viral variability and improving overall immunogenicity.

## Electronic supplementary material

Below is the link to the electronic supplementary material.

Supplementary Material 1

Supplementary Material 2

Supplementary Material 3

## Data Availability

No datasets were generated or analysed during the current study.
