# Supplementary figures and images for: Adenoviral fiber-knob based vaccination elicits efficient neutralizing antibodies and T cell responses against adenovirus infection
Source: Virol J. 2024 Oct 7;21:246. doi: 10.1186/s12985-024-02520-w (PMC11457358; doi:10.1186/s12985-024-02520-w)

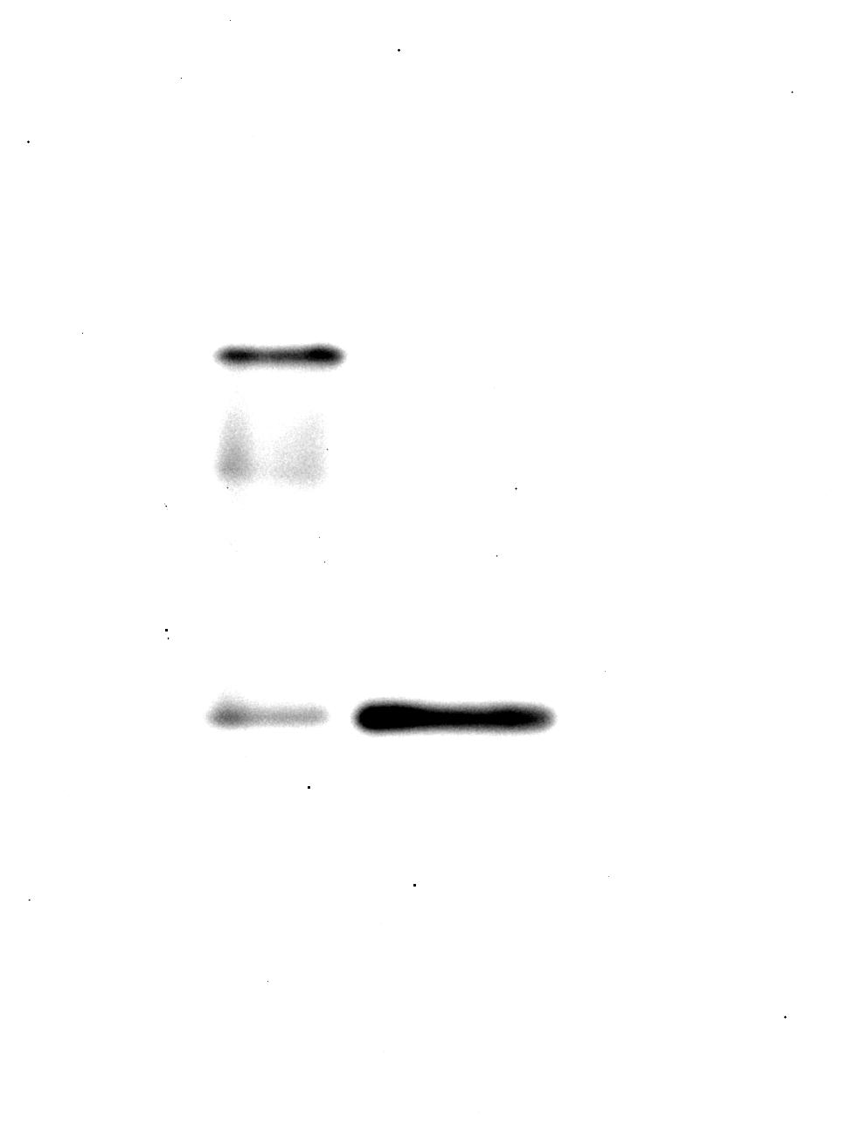

Supplement: Supplementary file 1 — Supplementary Material 1 [file 12985_2024_2520_MOESM1_ESM.tif]

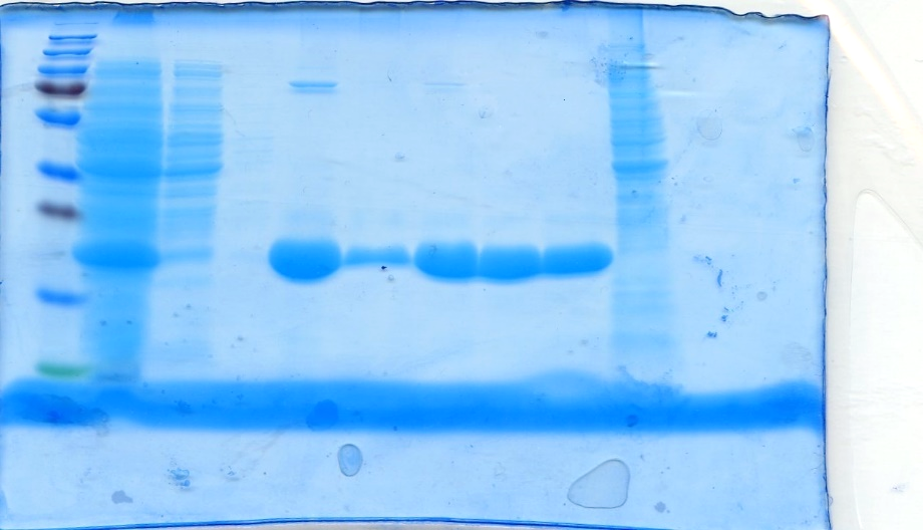

Supplement: Supplementary file 2 — Supplementary Material 2 [file 12985_2024_2520_MOESM2_ESM.tif]

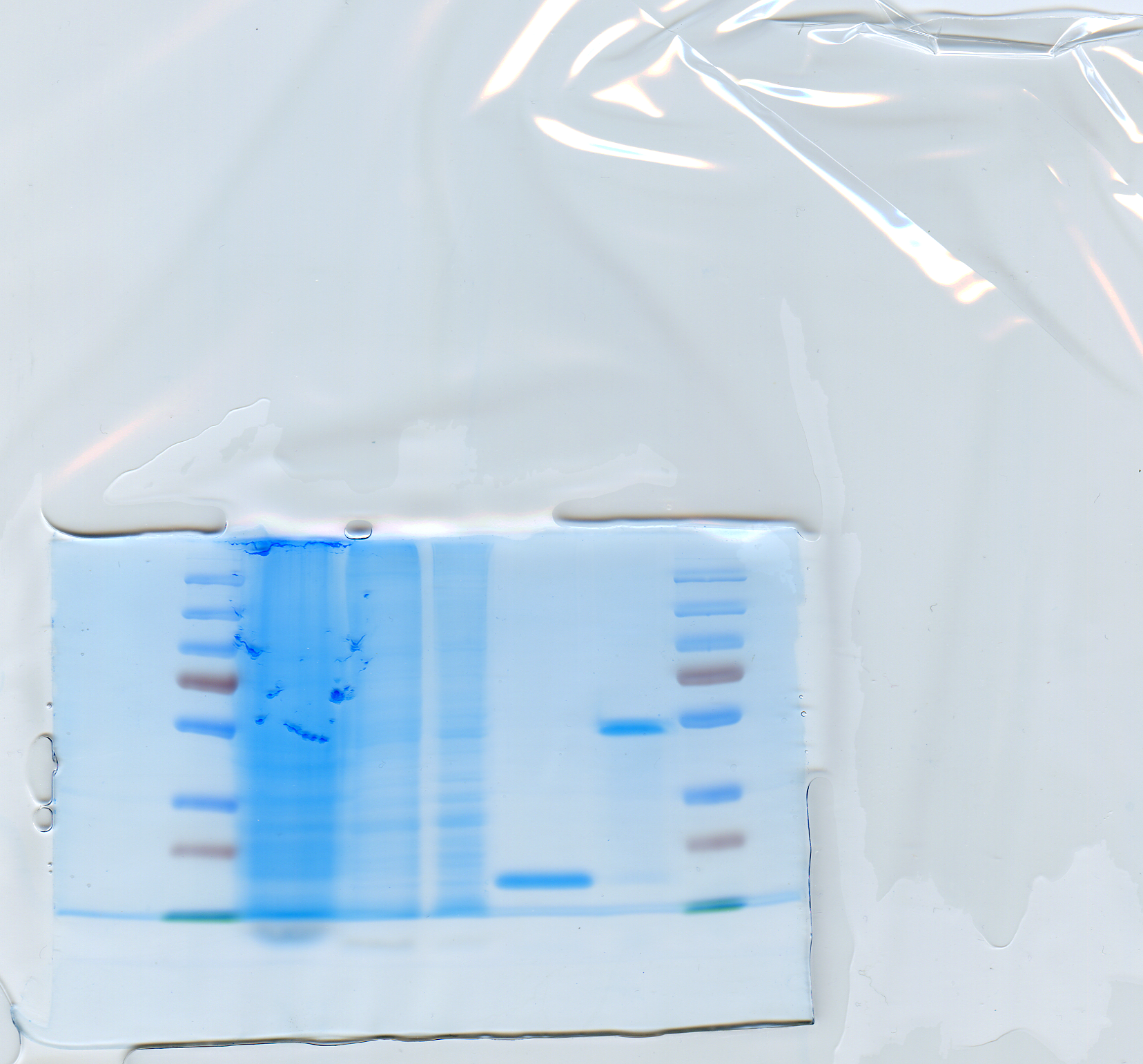

Supplement: Supplementary file 3 — Supplementary Material 3 [file 12985_2024_2520_MOESM3_ESM.tif]
